# Evaluation of the multidrug-resistant tuberculosis surveillance system in Maputo City, Mozambique in the period 2017-2018

**DOI:** 10.11604/pamj.2022.41.284.30611

**Published:** 2022-04-07

**Authors:** Dionísia Alfredo Balate, Ivan Manhiça, Bachir Macuacua, Benedita José, Denise Banze, José Carlos Langa, Cynthia Semá Baltazar, Jahit Sacarlal, Erika Valeska Rossetto, Celso Khosa

**Affiliations:** 1 Field Epidemiology Training Program, Maputo, Mozambique,; 2 National Tuberculosis Control Program, Maputo, Mozambique,; 3 Instituto Nacional de Saúde, Marracuene, Mozambique,; 4 Faculty of Medicine, Universidade Eduardo Mondlane, Maputo, Mozambique,; 5 MassGenics, Assigned to Centers for Disease Control Prevention, Maputo, Mozambique

**Keywords:** Tuberculosis, multidrug-resistant, public health surveillance, Mozambique

## Abstract

**Introduction:**

multidrug-resistant tuberculosis (MDR-TB) remains a public health problem worldwide. In Mozambique, cases of MDR-TB have increased annually. In 2018, 1,206 cases were reported, as compared to 943 cases in 2017. The aim of this study was to assess the surveillance system for multidrug-resistant tuberculosis in Maputo City.

**Methods:**

an extract from the national database was considered for a cut-out of the City of Maputo in the period 2017-2018; the study was conducted per the guidelines of the Centers for Disease Control and Prevention, where the description of the system was carried out, and evaluation of the attributes. Each attribute was evaluated according to the established criteria and parameters.

**Results:**

the surveillance system is based on the collection of data in health centers. Four hundred and six cases of MDR-TB were notified, of which 56.8% (231/406) were male and 95.9% (386/406) were ≥15 years. The system was complex with 4 levels of information transmission. With regard to flexibility, there was no changing the variables in the database. Acceptability was good. The quality of the data was regular with discrepancy of data of 14.5%. The system was considered stable as there was no system interruption. Timeliness with case notification monthly. The system sensitivity was 72.9%, the positive predictive value (PPV) was 2.3% and regarding utility the system has fulfilled its objectives.

**Conclusion:**

the system was not flexible, the data quality was regular, had moderate sensitivity and low positive predictive value. Continuous assessment of data and scale up the diagnosis for the detection of cases of MDR-TB is recommended.

## Introduction

Tuberculosis (TB) is a communicable disease that is a major cause of health problems, one of the top 10 causes of death in the world and the leading cause of death from a single infectious agent above human immunodeficiency virus (HIV) [1]. Globally, an estimated 10.0 million people fell ill with TB in 2018 of which 24% were located in Africa [1].

According to the World Health Organization (WHO), Mozambique is part of 14 countries that are simultaneously in 3 groups of countries with a high burden of TB, TB/HIV and MDR-TB and also of the very few countries in the world with an incidence above 500 per 100,000 inhabitants. In 2018, the country reported 1,206 cases of MDR-TB where the Maputo City was part of the 6 provinces that together notified about 81% of the cases notified in the country [2]. Of the total number of MDR-TB patients evaluated, the treatment success rate was 49%, below the world average, which is 55%. The death rate was 21%, much higher than what is expected of <5% [2].

Public health surveillance is the continuous and systematic collection, analysis, interpretation and dissemination of data related to a health event for public health action to reduce morbidity and mortality and improve the health of populations [3]. WHO argues that the data cannot accurately describe the TB load in a country if the surveillance system collects incomplete, inconsistent or incorrect information [4]. The ability of surveillance systems to accurately describe disease patterns is of public health importance [5].

In Mozambique, the National Tuberculosis Control Program (NTCP) is the entity responsible for controlling and eliminating tuberculosis in the country, through the implementation of various activities conceived in the national strategic TB plan (2014-2018) and which are aligned with the global strategies disease elimination [6]. A reliable information system is the essential basis for tuberculosis surveillance and control, both from an epidemiological and operational point of view [7].

Public health surveillance systems should be evaluated periodically as decision making depends on the timely availability of reliable data [8,9]. The aim of this study was to evaluate the surveillance system for multidrug-resistant tuberculosis in the City of Maputo in the period 2017-2018.

## Methods

We evaluated the MDR-TB surveillance system in Maputo City from January 2017 to December 2018 based on the guidelines of the Centers for Disease Control and Prevention, where qualitative (simplicity, flexibility, acceptability and data quality) and quantitative (representativeness, stability, opportunities, sensitivity and positive predictive value) attributes were evaluated based on the established parameters (Table 1), and the system's usefulness.

**Table 1:** criteria and parameters used to evaluate the attributes

Attribute	Rating criteria	Parameter	Values achieved	Score
Simplicity	Number of variables in the database	≤ 50 variables = 1	33 variables = 1	
		≤ 50 variables = 0		Score
	Need for training to use the system	Required = 0	Not required = 1	0 to 4 points
		Not required = 1		Simple: ≥ 3 points
	Method of transmission of data and information	Off line = 0	Offline = 0++	Complex: <3 points
		Online = 1		Achieved: 2/4
	Levels of sending information	Up to 3 levels = 1	4 levels = 0	Classification
		˃ 3 levels = 0		Complex
Flexibility	Introduction of new variables in the database	Introduced = 1	Without introducing new variables = 0	Score: 0 to 1 point
		Not introduced = 0		Flexible =1 point; not flexible = 0 point
				Achieved: 0/1 point
				Classification: not flexible
Acceptability	Completeness of fields	≥ 75% = good = 2	Completeness 2017 = 99.5%	Score
		51% to 74% = regular = 1	Completeness 2018 = 89.5%	0 to 2 points
		<50% bad = 0	Average = 94.5% = 1	Good acceptability = 2 points; regular acceptability = 1
	District participation rate		All districts had field completion above 75% = 1	Bad acceptability = 0
				Achieved: 2/2 points
				Classification: good acceptability
Data quality	Completeness of fields (name, district, US, sex, HIV tested, start of ART)	≥ 75% = good =2	Completeness 2017 = 99.5%	Score: 0 to 2 points
		51% to 74% = regular =1 <50% = bad = 0	Completeness 2018 = 89.5%	Good quality = 2 points
	Consistency between databases and existing data in the SIS-MA	Deviation of <10% - good quality data = 2	Average = 94.5% = 1	Regular quality = 1 point; bad quality = 0 point
		Deviation between 10% to 20% - medium quality data = 1	Deviation 2017 = 8%	Achieved: 1/2 point
		Deviation of> 20% - low quality data = 0	Deviation 2018 = 21%	Classification: regular quality
			Average = 14.5% = 0; total data quality 52.4% = 1	
Representativeness	Collection of mandatory epidemiological variables (time, place, person)	> 85% = high representativeness = 1	100% = 1	Score: 0 to 1 point
				Representative = 1 point
		<85% = low representativeness = 0		Low representativeness = 0; achieved: 1/1 point
				Classification: representative
Stability	System continuity	System not interrupted = stable = 1	System continuity = 1	Score: 0 to 1 point
		System interrupted = not stable = 0		Stable = 1 point
				Not stable = 0; achieved: 1/1 point
				Classification: stable
Opportunity	Notification of cases to central level	Monthly = timely = 1 More than 1 month = not timely = 0	Monthly = 1	Score: 0 to 1 point
				Timely = 1 point
				Not timely = 0 point
				Achieved: 1/1 point
				Classification: timely
Sensitivity	Proportion of MR-TB cases detected	≥90% = hight = 2	Sensitivity 2017 = 55.9% (146/261)	Score: 0 to 2 points; hight = 2 point
		≥70 and ≤89% =moderate = 1	Sensitivity 2018 = 89.9% (260/289)	
		<70%= low = 0	Average = 72.9% = 1	Moderate = 1 point; low = 0 point
				Achieved: 1/2 point
				Classification: moderate sensitivity
PPV	Cases notified to the surveillance system; laboratory closed with positive result	≥ 70% = high PVP = 1	VPP 2017 146/7064 * 100 = 1.8%; 2018 VPP 260/9111 * 100 = 2.8%	Score: 0 to 1 point; high PVP = 1 point
		<70% = low PVP = 0	Average = 2.3% = 0	Low PVP = 0 point
				Achieved: 0/1 point
				Classification: low PVP

The resistant tuberculosis surveillance system database was used as the data source. This contains 33 variables, distributed in 5 fields: 1) identification; 2) categories of patients; 3) test results; 4) treatment administered; and 5) treatment outcome. Patients of all ages registered in the resistant tuberculosis surveillance system database participated in the study. Maputo City was selected because in 2017 reported the highest number of cases and in 2018 it was part of the 6 provinces that together accounted for 81% of resistant TB cases.

**Data analysis:** a descriptive analysis of the data was performed using the Microsoft Excel 2019® and, as secondary data exist in the NTCP, ethical approval was not required as de-identified datasets were used.

The following criteria were used to evaluate the attributes: in simplicity we evaluated: (a) number of variables in the database; (b) the need to train human resources; (c) information transmission method; (d) levels of sending information. Flexibility was assessed by checking the change in variables in the database and the ability of the system to adapt to changes. For acceptability we calculated: (a) the completeness of the fields; (b) district participation rate in filling in the fields. In the quality of the data, we verified: (a) completeness of the fields of variables: sex, age, health unit, district, HIV test and initiation of antiretroviral treatment (ART); (b) consistency of the data where the data from the Excel spreadsheet and the Health-Monitoring and Evaluation Information System (SIS-MA) were compared for the following variables: sex, age group, notified MDR-TB cases, HIV-tested patients, TB/HIV and TB/HIV patients on ART where the deviation was calculated using the following formula:


$$\frac{\text{Recounted data in the period under review - Data reported in the period under review}}{\text{Recounted data in the period under review}}\text{×100}$$

In the representativeness, the presence of epidemiological data (person, time and place) was verified, where: (a) Person - distribution of cases by sex and age group; (b) time - MDR-TB cases according to the year of diagnosis; (c) place - cases of MDR-TB by source. In terms of stability, the continuity of the system's functioning was verified through the description of the MDR-TB cases notified by health unit and by month. Timeliness, on the occasion, the frequency of notification of cases to the central level was verified. For sensitivity, the proportion of cases notified by the surveillance system was calculated using the formula:


$${\text{Sensitivity}}_{\text{MDR-TB}}\text{=}\frac{\text{MDR-TB cases reported}}{\text{Expected MDR-TB case}}\text{×100}$$

Positive predictive value (PPV), the positivity rate of cases was calculated using the formula:


$${\text{PPV}}_{\text{MDR-TB}}\text{=}\frac{\text{Total number of confirmed cases MDR-TB using GeneXpert test}}{\text{Total of cases submitted to the laboratory for GeneXpert test}}\text{ ×100}$$

**System utility:** the capacity of the system to meet the objectives was verified.

## Results

**Figure 1:**
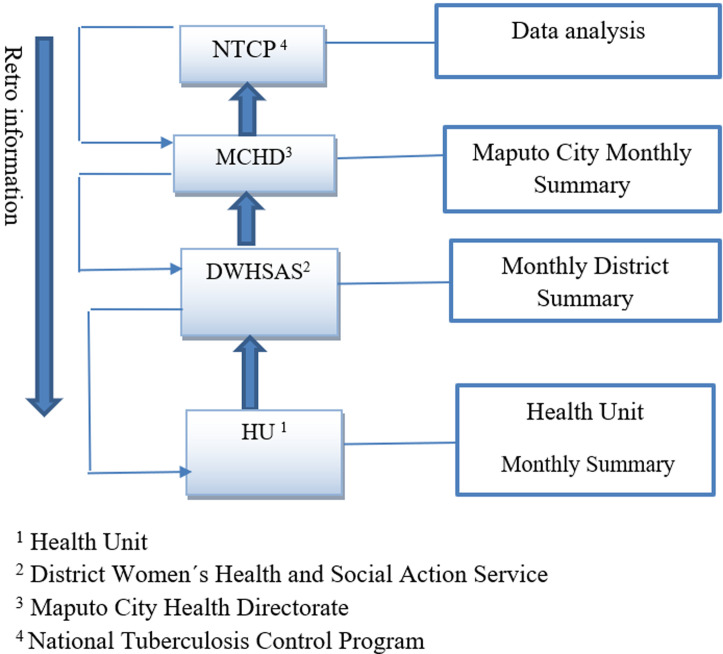
information flow of multidrug-resistant tuberculosis in Maputo City

**System description:** the MDR-TB surveillance system in Maputo City is structured and based on data collection at the health centers level, which are the basic management unit for MDR-TB, patients are registered, treated, and followed at this level, these are part of the NTCP. The information is initially recorded in the MDR-TB patient record book that contains identification (demographic data), patient categories, treatment administered, test and treatment results. A patient treatment card is also filled out, with information for each visit. The basic analysis of the data is made at the health unit and is compiled monthly on the MDR-TB monthly notification form. These data are sent to the district, where the district aggregates and makes the monthly district summary and sends it to the Maputo City Health Directorate (MCHD). In turn, MCHD aggregates data and summarizes data for the entire city and sends it to the NTCP in an Excel spreadsheet (the nominal list and the monthly summary). After receiving the information, the NTCP analyzes the data and sends the retro information following the flow backwards (Figure 1).

### System attributes

**Simplicity:** the Excel spreadsheet (the nominal list) contains 33 variables, there is no need of extensive training to complete it, the method of transmitting information is offline and is done at 4 levels (health unit, district, MCHD and NTCP). For a system to be considered simple, it must not contain more than 3 levels. In this context, the system was classified as complex.

**Flexibility:** we found that there was no change in variables in the database and that the database is a copy of the record book that is in the health unit. The system was classified as not flexible.

**Acceptability:** the completion of the fields in 2017 was 99.5% and 89.5% in 2018, with an average completion rate of 94.5%. In the completeness assessment by district, it was observed that all districts participated with a field filling rate above 75%, and in 2017, the district of Kamavota and Katembe had 100% and in 2018 the district of Kamaxakene had a greater participation with 93.1%. The system was classified as good acceptability.

**Data quality:** in 2017, a total of 146 patients were registered, a total of 1,022 fields were evaluated. In 2018, 260 patients were registered, a total of 1,820 fields were evaluated. The start variable of antiretroviral treatment was the least filled in the two years with 96.0% in 2017 and 63.0% in 2018. The overall average of the completeness of the fields for the 2 years was 94.5%. When comparing the data from the Excel and SIS-MA spreadsheet, there was inconsistency in the data, where the data deviation in 2017 was 8.0% and in 2018 it was 21.0%. The overall average deviation of the data was 14.5% (Table 2). The value obtained in completeness and in the deviation divided by 2 was added and the general average of the data quality was 52.4%. The data quality was regular.

**Table 2:** comparison between SIS-MA data and Excel spreadsheet data

Variables	2017		Deviation	2018		Deviation	Deviation 2017/2018
	Excel spreadsheet	SIS-MA		Excel spreadsheet	SIS-MA		
Male	83	80	4%	148	107	28%	16%
Women	63	73	16%	112	76	32%	24%
Age range	≥15	≥15	0%	≥15	≥15	0%	0%
Notified cases	146	153	5%	260	183	30%	18%
HIV-tested patients	141	150	6%	237	208	12%	9%
HIV positive patients	137	100	27%	137	128	7%	17%
TB/HIV patients who started ART	95	95	0%	94	128	36%	18%
Total			8%			21%	14.5%

**Representativeness:** a total of 406 cases of MDR-TB were reported, of which 146 in 2017 and 260 in 2018. The predominant age group was ≥15 years with 97.9% (143/146) in 2017 and 93.4% (243/260). In 2017, the age of cases ranged from 1 to 84 years with a median of 34 years and in 2018 the range was from 1 to 79 with a median of 33 years. Male gender was more prevalent with 56.8% (83/140) in 2017 and 56.9% (148/260) in 2018. For the two years the cases were frequently from the Kamavota District with 31.5% (46/146) cases in 2017 and 37.6% (98/260) in 2018. The system was representative.

**Stability:** during the two years, the TB-MR surveillance system in the City of Maputo collected and provided data from all health units that record, treat and monitor cases without any interruption, the surveillance capacity was maintained. Mavalane Health Center was the one that reported the most cases in the two years with 18.4% (27/146) cases in 2017 and 20.3% (53/260) in 2018. Depending on the time, every month there was notification of cases to the central level as recommended by the surveillance system, where in 2017 most cases were reported in July with 12.3% (18/146) cases and in 2018 it was in November with 15.0% (39/260) cases. The system was stable.

**Timeliness:** being the timeliness the speed between the various steps in a public health surveillance system, we found that the frequency of notification of cases MDR-TB to the central level is monthly. The system was assessed as timely.

**Sensitivity:** we found that in 2017, of the 261 expected cases of MDR-TB, the surveillance system notified 55.9% (146/261), and in 2018 of the 289 expected cases, the system notified 89.9% (260/289). The average sensitivity was 72.9%. The system was classified as having a moderate sensitivity. The Positive Predictive Value (PPV) in 2017 was 1.8% and in 2018 it was 2.8%. The average PPV was 2.3% and classified as low.

**Utility:** the surveillance system of the MDR-TB proved to be useful for achieving its objectives because it provides data for understanding the magnitude of the distribution and trends over time, providing subsidies for control actions.

## Discussion

The flow of information presented in this study shows that the MDR-TB surveillance system in the City of Maputo is well structured with well-defined levels of responsibilities. Regarding the attributes, although there is no need of extensive training of human resources, the system was complex, influenced by the presence of 4 levels of information transmission. For a system to be considered simple, it must not contain more than 4 levels [10]. Several results about simplicity were found in evaluations made in other countries, where in Brazil in the evaluation made by Tourinho 2020 and Oliveira 2010 the system was complex [11,12], in Yemen and South Africa the system achieved average simplicity [13,14] and in Ghana it was simple [15].

Since the database is a copy of a register book, to change variables in the database, it would be necessary to review the primary source, which is the register book found in the health unit, in this context it would be necessary to train providers, for updating new variables and printing new registration books for all health units, which may require financial resources, and the consequent delay in implementing changes, which made the system not flexible. The acceptability of the system was good, which shows the commitment of the actors involved with the activities of the system. Similar results were found in Nigeria [5]. The MDR-TB surveillance system describes the cases by sex, age and origin, this yields knowledge of the characteristics of the population affected by the disease. Contrary results were found in Ghana where the system was not representative [15].

The quality of the data was regularly influenced by the inconsistency of the data between the Excel spreadsheet and SIS-MA. The data discrepancy problem was also found in South Africa [15]. In Yemen the comparison of data in the case files and in the registry showed no discrepancies [14]. As for the sensitivity, although there was an increase in the notification of cases, the two years failed to reach the established goal, this fact may be demonstrating the challenge that the system has in the notification of cases. Nevertheless, it is important to note that the expected case by year might have been overestimated. The average PPV was 2.3%, this value indicates that the positivity rate among suspected patients is low, however, the system may be testing many cases without criteria for testing, on the other hand it may be a positive point, as this is less likely to lose cases. Regarding utility, the system proved to be useful due to its ability to register diagnostic and control of MDR-TB, similar results were found in Yemen [14] a study in Ghana showed that the system was useful although it only partially fulfilled its objectives [16].

## Conclusion

The system had good acceptability, representative, timely and stable. However, it was limited for the following criteria: complex, not flexible, regular data quality, moderate sensitivity and low PPV. It is recommended continuous monitoring and evaluation of data to improve quality, for the notification of cases, the identification of suspected cases at the health unit level should be intensified, and the NTCP should scale up the diagnosis to detect cases of MDR-TB.

### What is known about this topic

- *MDR-TB surveillance data are important for decision making;*
- *Surveillance data must meet established goals and quality standards*.

### What this study adds

- *The national MDR-TB surveillance system of Mozambique is well structured;*
- *System stakeholders are committed to the system;*
- *The MDR-TB surveillance system has gaps in data it is not easy to operate, not flexible, moderate sensitivity, and has low PPV*.

## References

[1] World Health Organization Global tuberculosis report 2018.

[2] World Health Organization (2019). Global tuberculosis report 2019.

[3] Centers for Disease Control and Prevention Updated guidelines for evaluating public health surveillance systems.

[4] World Health Organization (2014). Understanding and using tuberculosis data.

[5] Kwaghe AV, Umeokonkwo CD, Aworh MK (2020). Evaluation of the national tuberculosis surveillance and response systems, 2018 to 2019: national tuberculosis, leprosy and buruli ulcer control programme, Abuja, Nigeria. Pan African Medical Journal.

[6] Jiang WX, Huang F, Tang SL, Wang N, Du X, Zhang H (2021). Implementing a new tuberculosis surveillance system in Zhejiang, Jilin and Ningxia: improvements, challenges and implications for China´s national health information system. Infect Dis Poverty.

[7] European Centre for Disease Prevention and Control (ECDC) (2014). Data quality monitoring and surveillance system evaluation: a handbook of methods and applications. ECDC Technical Document.

[8] Alemu T, Gutema H, Legesse S, Nigussie T, Yenew Y, Gashe K (2019). Evaluation of public health surveillance system performance in Dangila District, Northwest Ethiopia: a concurrent embedded mixed quantitative/qualitative facility-based cross-sectional study. BMC Public Health.

[9] World Health Organization UNAIDS & UNAIDS/WHO Working Group on Global HIV/AIDS and STI Surveillance (2013). Evaluating a national surveillance system.

[10] Mota DM, Freitas DRC, de Araújo WN (2012). Evaluation of the system of sanitary vigilance of blood at the federal level, Brazil, 2007. Cien Saude Colet.

[11] Tourinho BD, Oliveira PB, Silva GDMD, Rocha MS, Penna EQAA, Pércio J (2020). Evaluation of the drug-resistant tuberculosis surveillance system, Brazil, 2013-2017. Epidemiol Serv Saude.

[12] Oliveira PB, Oliveira GP, Codenotti SB, Nóbrega AA (2010). Assessment of the tuberculosis surveillance system in Rio de Janeiro, Brazil, 2001 to 2006. Cad Saùde Colet.

[13] Abdulmughni J, Mahyoub EM, Alaghbari AT, Al Serouri AA, Khader Y (2019). Performance of multidrug-resistant tuberculosis surveillance in Yemen: interview study. JMIR Public Health Surveill.

[14] Heidebrecht CL, Tugwell PS, Wells GA, Engel ME (2011). Tuberculosis surveillance in Cape Town, South Africa: an evaluation. Int J Tuberc Lung Dis.

[15] Frimpong-Mansoh RP, Calys-Tagoe BNL, Therson-Coffie EF, Antwi-Agyei KO (2018). Evaluation of the tuberculosis surveillance system in the Ashaiman municipality, in Ghana. Pan African Medical Journal.

